# Domestic

**Published:** 1840-07-25

**Authors:** 


### DOMESTIC.

*Report of a case in which a Testis was found in the Abdomen of a man post mortem.* By George W. Bayless, M. D.—In making an autopsical examination, at the Louisville Marine Hospital, May 23d, 1840, of the body of Dunham, a boatman, aged twenty-seven years, large and muscular, we found in the left iliac region, about an inch and a half from the internal abdominal ring, a testis, about one inch in length, three-fourths of an inch in breadth, and half an inch thick. From an inspection of its exterior, its organization seems to be perfect. We made no dissection, being desirous of preserving it entire *in situ*. It has a complete peritoneal investment, and is attached by a peduncle of this membrane to the walls of the abdomen, obliquely upwards and backwards from the ring. It hangs loosely in the cavity of the abdomen, and seems to have been arrested in its descent, by an adhesion of, what would have been, if it had descended into the scrotum, its anterior convex edge, to the side of the fundus of the bladder. The adhesion is firm, and almost ligamentous in its appearance; and there being no trace whatever of recent inflammation, it is fairly inferrible, that it was contracted when the testis was in its passage from its original situation to the scrotum. This supposition is strengthened by the fact, that the epididymis is drawn off about three-fourths of an inch from the body of the testis, seemingly by the contraction of the gubernaculum testis, which is firmly adherent to it. This last fact, the separation of the epididymis from the body of the testis, not only renders it probable that the descent of this organ was arrested by a mechanical cause, as the adhesion above mentioned, but it also strengthens the supposition of the contractility of the gubernaculum, by which the transition of the testis is thought to be effected. The vas deferens leading from it is about the same size and appearance as that of the other side, and takes its ordinary course by the side of the bladder to its vesicula seminalis, which is about a third or half an inch shorter than its fellow of the opposite side, and is also only about two-thirds of its breadth. The internal abdominal ring, canal, and external ring are large, permitting, without difficulty, the passage of the little finger, and terminating in a *cul de sac* lying within the scrotum, about two inches long and an inch and a half wide. Hernia seems to have been prevented by the testis' covering the mouth of the internal ring, its peritoneal peduncle being sufficiently long to allow its assuming that position, where, indeed, it was found, and seems most inclined to lie.

All the corresponding parts of the opposite side are perfect in their organization and development, and all occupy their ordinary situations.

No history of the individual could be obtained, but from his athletic form, and the full development of his generative organs, there can scarcely arise a suspicion, that impotency existed in his case. This fact possesses some value in its bearing upon legal medicine, and as connected with the question of castration, which sometimes comes up in courts of justice.

The specimen has been placed in the Pathological Cabinet of the Medical Institute of Louisville, where it may be seen.—*Western Journal of Medicine and Surgery, June*, 1840.

